# A Clinical Odyssey Involving Cleidocranial Dysplasia: Report of a Rare Case

**DOI:** 10.7759/cureus.51024

**Published:** 2023-12-24

**Authors:** Nishu Agarwal, Pallavi Daigavane, Ranjit Kamble, Dhwani Suchak

**Affiliations:** 1 Orthodontics and Dentofacial Orthopaedics, Sharad Pawar Dental College and Hospital, Datta Meghe Institute of Higher Education and Research, Wardha, IND

**Keywords:** cleidocranial, multidisciplinary approach, orthodontic perspective, skeletal manifestations, cranial anomalies

## Abstract

Cleidocranial dysplasia (CCD) is a rare genetic disorder that causes cranial and skeletal abnormalities. This case report presents a comprehensive analysis of a rare instance of CCD, highlighting its clinical manifestations through an orthodontic lens shedding light on the challenges and complexities associated with managing this uncommon condition. The patient, an 18-year-old female, presented with a variety of symptoms, including delayed eruption of permanent teeth, abnormal facial features, and prominent cranial abnormalities. Multiple teeth in both the arches were missing including over-retention of primary teeth. Features of cleidocranial dysplasia were evident in her facial appearance. Treatment of CCD requires a multifaceted approach, often involving orthodontic interventions, dental extractions, and corrective surgeries to address cranial deformities and other skeletal anomalies. The report emphasizes the importance of multidisciplinary collaboration in diagnosing and managing such cases, shedding light on the distinctive features of CCD and their implications for orthodontic treatment on what kind of best treatment can be given to these patients. This case serves as a reminder of the importance of raising awareness about rare genetic disorders like CCD, as early diagnosis and intervention can significantly improve the patient's quality of life. Furthermore, it underscores the significance of a collaborative and holistic healthcare approach in managing such complex conditions. It emphasizes the need for continued research, awareness, and support for individuals affected by such conditions.

## Introduction

Cleidocranial dysplasia (CCD) is an inherited autosomal dominant skeletal disorder [1]. It can also occur spontaneously. Sainton and Marie were the first to report on it in 1898 [2]. CCD is also known as mutational dysostosis or Marie and Sainton's disease. CCD affects both sexes equally and has a prevalence of one per one million people; it tends to pass down through generations [3].

This condition is characterized by skeletal abnormalities affecting bones and teeth due to mutations in the RUNX2 gene [4]. Mesenchymal condensation, osteoblast differentiation from mesenchymal stem cells, chondrocyte hypertrophy, and vascular invasion in the growing skeleton are further processes that depend on the gene [5,6]. The diseased gene has been identified on chromosome 6p21 within a region containing CBFA1, a transcription factor from the runt family [7].

Among its many manifestations, delayed or incomplete eruption of permanent teeth stands out as a prominent dental concern for these patients. Understanding the intricacies of this genetic disorder is crucial for clinicians and orthodontists to provide comprehensive and patient-centered care. The importance of orthodontic treatment is in guiding tooth eruption, aligning teeth, and correcting bite issues. Such a treatment should have a positive impact on speech, aesthetics, and overall oral health for individuals living with CCD. A multidisciplinary approach is necessary for managing CCD patients effectively [8].

## Case presentation

**Figure 1:**
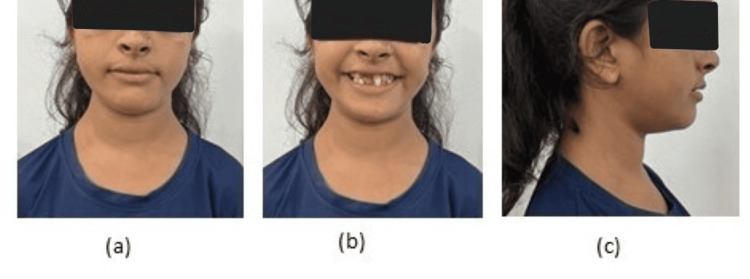
Extraoral photographs (a) frontal non-smiling (b) frontal smiling (c) profile

An 18-year-old diagnosed with CCD came to the Department of Orthodontics and Dentofacial Orthopedics with the chief complaint of multiple missing teeth and poor esthetics. There was no positive medical history. Family history was also negative. The patient gave the history of visiting a private clinic three years back where no treatment was given to her and the patient was kept under observation for the normal eruption of permanent teeth. She exhibited classic cleidocranial dysplasia characteristics such as hypertelorism, a broad forehead, and a short upper third of the face with a straight profile (Figure 1).

**Figure 2:**
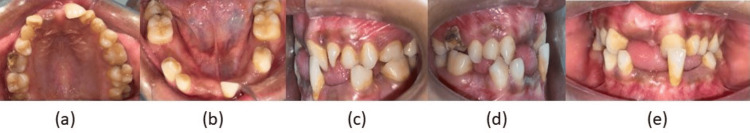
Intraoral photographs (a) maxillary arch (b) mandibular arch (c) right molars in occlusion (d) left molars in occlusion (e) anterior teeth in occlusion

The initial intraoral examination revealed a class III molar relationship, anterior crossbite, and multiple missing permanent teeth with four over-retained primary teeth, i.e., 53, 63, 74, and 83 (Federation Dentaire Internationale numbers) (Figure 2).

**Figure 3:**
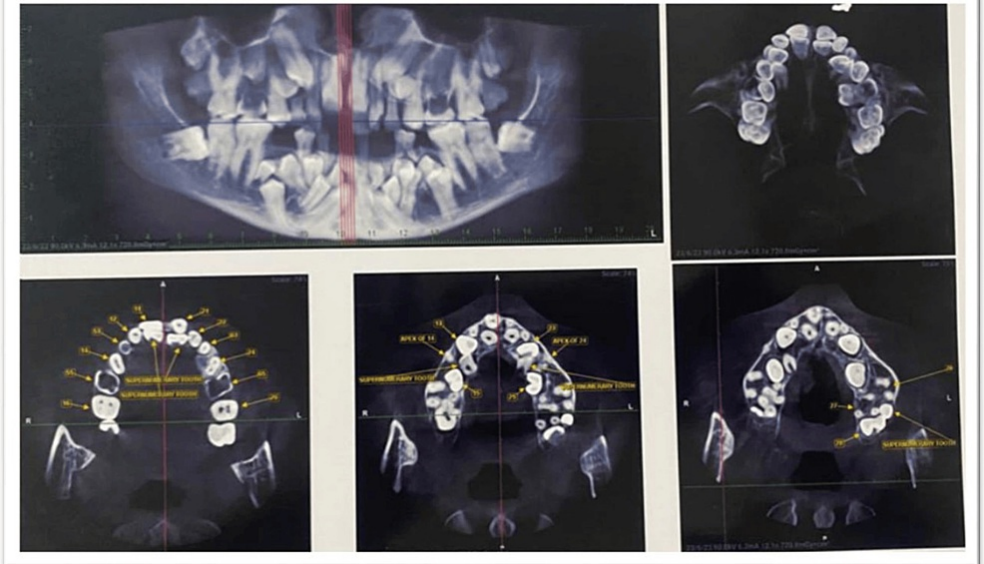
CBCT images CBCT: cone beam CT

Radiographic examination on cone beam CT (CBCT) revealed 12 impacted permanent teeth, i.e., 13, 15, 23, 25, 34, 35, 41, 32, 42, 33, 43, and 45, and a total of five impacted supernumerary teeth in the maxillary arch and 11 in the mandibular arch (Figure 3).

This report aims to provide insights into the diagnostic process, clinical manifestations, and orthodontic considerations in managing a rare case of CCD. Both written and informed consent were taken from the patient.

Treatment plan

A multidisciplinary approach involving orthodontists, oral surgeons, and a prosthodontist was employed to address the patient's complex condition. To induce eruption, the most promising treatment involves surgical exposure of the unerupted permanent teeth, surgical removal of supernumerary teeth, and orthodontic intervention. Orthodontic treatment planning focuses on creating space for the eruption of permanent teeth, considering the retained deciduous teeth and surgical exposure of impacted permanent teeth along with surgical extractions of multiple impacted supernumerary teeth. These extractions and exposure of permanent teeth are to be done under general anesthesia archwise. All the permanent teeth will be guided into occlusion by orthodontic traction force. Another treatment plan could be the extraction of some or all erupted and unerupted teeth, followed by the construction of full or overlay dentures, but there is quite a possibility of extensive alveolar bone loss if teeth are extracted. Settling of the occlusion will be done after bringing the impacted teeth into the arch by traction force.

## Discussion

CCD's most distinguishing dental features are retained primary teeth and multiple supernumerary teeth as seen in this case. According to a study conducted by Cheng et al., the number of supernumerary teeth increases with age in patients with CCD [9]. Primary tooth retention, skeletal class III malocclusion, midfacial hypoplasia, and overclosure, all cause functional and aesthetic issues [10].

Individuals who are diagnosed with CCD require prolonged orthodontic as well as surgical treatments, making dental management difficult. An interdisciplinary team must carefully plan and carry out orofacial findings in CCD. The course of treatment can last for a very long time and is typically not finished until growth has stopped. For patients with CCD, recent advancements in dentistry have improved treatment options and results. Dental implants, for instance, have shown to be effective for replacing teeth that cannot be orthodontically guided into the arch as well as for minimally invasive orthodontic tooth movement [11]. In a study conducted by Ahmad et al., full mouth rehabilitation was done using basal implants in patients with CCD. Though osteoblastic activity is compromised in these patients, osteointegration and stability of implants were reported in their study [12].

Orthognathic surgery corrects CCD skeletal deformity while reducing the need for compensation treatment. As a result, while this approach achieves the best treatment outcomes in the shortest amount of time, patients must undergo orthognathic surgery, which is expensive. Patients with CCD may also benefit from prosthetic treatment. Atil et al. described instances of middle-aged CCD patients receiving oral rehabilitation using implant-supported fixed dental prostheses [13]. In cases of multiple impacted teeth in patients with CCD where there is a risk of fracture of bone or risk of damage to the nerve, a removable prosthesis can be given to the patients to restore function [14]. While avoiding the risks of surgery and orthodontic treatment, prosthetic treatment can quickly restore the appearance and functionality of the mouth.

## Conclusions

It is essential to provide early diagnosis and comprehensive care to individuals with CCD to address their unique needs and improve their quality of life. Orthodontic treatment is an indispensable component of the multidisciplinary approach to managing CCD. By offering personalized treatment plans, early intervention, and a comprehensive approach, we can empower CCD patients to overcome dental challenges, improve their oral health, and embrace a brighter and more confident future. Beyond the functional benefits, orthodontic treatment in CCD patients can lead to improved speech and enhanced psychological well-being. A confident smile and improved oral function can positively impact the overall quality of life for individuals living with this rare genetic disorder.
